# The impact of mitochondrial function/dysfunction on IVF and new treatment possibilities for infertility

**DOI:** 10.1186/1477-7827-12-111

**Published:** 2014-11-24

**Authors:** Heide Schatten, Qing-Yuan Sun, Randall Prather

**Affiliations:** Department of Veterinary Pathobiology, University of Missouri, Columbia, MO USA; State Key Laboratory of Reproductive Biology, Institute of Zoology, Chinese Academy of Sciences, 100080 Beijing, China; National Swine Resource and Research Center, University of Missouri, 65211 Columbia, USA; Division of Animal Science, University of Missouri, 65211 Columbia, USA

**Keywords:** Mitochondria, Oocytes, Embryos, Embryo development, Metabolism, In vitro fertilization, Mitochondrial supplementation, Infertility treatment

## Abstract

Mitochondria play vital roles in oocyte functions and they are critical indicators of oocyte quality which is important for fertilization and development into viable offspring. Quality-compromised oocytes are correlated with infertility, developmental disorders, reduced blastocyst cell number and embryo loss in which mitochondrial dysfunctions play a significant role. Increasingly, women affected by metabolic disorders such as diabetes or obesity and oocyte aging are seeking treatment in IVF clinics to overcome the effects of adverse metabolic conditions on mitochondrial functions and new treatments have become available to restore oocyte quality. The past decade has seen enormous advances in potential therapies to restore oocyte quality and includes dietary components and transfer of mitochondria from cells with mitochondrial integrity into mitochondria-impaired oocytes. New technologies have opened up new possibilities for therapeutic advances which will increase the success rates for IVF of oocytes from women with compromised oocyte quality.

**Electronic supplementary material:**

The online version of this article (doi:10.1186/1477-7827-12-111) contains supplementary material, which is available to authorized users.

## Background

Mitochondria play vital roles in oocyte functions and they are critical indicators of oocyte quality. Among the major mitochondrial functions is the production of adenosine triphosphate (ATP) to provide energy, but unlike in somatic cells, ATP in most mammalian oocytes is mainly generated through the glycolytic and Pentose Phosphate Pathway (PPP) rather than the oxidative phosphorylation (OXPHOS) pathway [1, 2] used by most differentiated somatic cells. Correlated with these metabolic differences are significant structural differences; since the embryo primarily uses glycolysis and PPP, the mitochondria are round and contain few cristae, while mitochondria using the OXPHOS pathway display elongated shapes and contain an elaborate system of cristae that distinguishes the inner mitochondrial membrane. Changes in mitochondrial structure from the round (immature) phenotype to the elongated (mature, differentiated) phenotype takes place during embryogenesis when cells differentiate and mitochondrial metabolism changes from primarily glycolysis to OXPHOS.

Compared to our knowledge of mitochondria in somatic cells, relatively little is known about mitochondria in oocytes and in the preimplantation embryo but it is well known that mitochondrial dysfunction in the oocyte and embryo play significant roles in infertility and in developmental abnormalities [3–7].

Fertility disorders have become a growing problem worldwide [8, 9], and mitochondrial dysfunction associated with infertility have clearly been shown in women affected by diseases or metabolic disorders such as diabetes and obesity [3, 10–12] as well as changes in metabolism resulting from oocyte aging [8, 11–17]. Other mitochondrial dysfunctions are still unexplained but have resulted in an increased number of women requiring in vitro fertilization (IVF) or other assisted reproductive technologies (ARTs). Currently, one percent of all babies in the Western world are already being produced through ART and the numbers are increasing reviewed in [8, 9, 18, 19].

The effects of mitochondrial dysfunctions as well as sub-optimal mitochondrial functions are correlated with meiotic spindle abnormalities. As will be addressed in more detail below, mitochondrial functions are important for the formation of meiotic spindles and for maintenance of the MII spindle before fertilization. Insufficient ATP generation will result in aneuploidy, a condition in which chromosomal segregation errors are frequently encountered. In human, oocyte chromosomal segregation is error-prone and these errors have been estimated to be about 15–20% [20] and 5% of all pregnancies are aneuploid [21]; reviewed in [19]. The frequency of aneuploidy increases significantly in oocytes from obese or diabetic females [3, 10–12] and in aging oocytes [8, 11, 12, 14, 15], as will be discussed in section 2. While cytoplasmic transfer from healthy donor oocytes with fully functional mitochondria has been successfully applied to overcome mitochondrial dysfunctions in quality-compromised oocytes [6, 8, 11, 12, 14, 15, 22, 23] the procedures raised concerns including ethical concerns, but new studies clarified the potential risks; such as introducing heteroplasmy or mitochondrial genetic disease [24]; reviewed in 6. These techniques have resulted in new possibilities to overcome mitochondrial dysfunction in quality-compromised oocytes [25–31]. The present review will address: 1) Mitochondrial structure, dynamics, characteristics, and their functions in oocyte maturation, fertilization and embryo development; 2) Mitochondrial dysfunction in quality-compromised oocytes; and 3) Possibilities to overcome mitochondrial dysfunctions in oocytes to improve IVF success rates.

## Section 1: mitochondrial structure, dynamics, characteristics and their functions in oocyte maturation, fertilization, and embryo development

Based on the significant need to increase success rates in IVF clinics new strategies are in demand to overcome mitochondrial dysfunctions, but before therapeutic interventions can be applied the metabolic activities and dynamics of mitochondria in unaffected oocytes and embryos need to be understood more fully. Many aspects of mitochondrial function and dysfunction are still unexplored in oocytes and preimplantation embryos and many questions remain to be answered. This section will address our current understanding of mitochondrial dynamics and highlight the aspects that still need clarification.

Mitochondria are multifunctional organelles with critical functions in cellular energy production, calcium homeostasis [32], cell signaling, apoptosis [33] and several other cellular processes [34]; reviewed in [35, 36]. Mitochondria dynamics and motility are correlated with mitochondrial functions and mitochondria have an enormous ability to modulate their functions, dynamic behavior, and metabolic activities, depending on different environmental conditions. Most studies on mitochondrial metabolism, dynamics, biochemistry, molecular composition and related functions have been performed in somatic cells in which mitochondrial dysfunctions have been correlated with specific structural and molecular abnormalities associated with diseases such as neurological disorders, heart disease, diabetes, and cancer; reviewed in [35, 36].

Mitochondria are semiautonomous organelles that contain their own genome of ca. 16.6 kb circular mitochondrial DNA (mtDNA), encoding for 13 essential protein subunits of complexes I, III, IV, and V of the respiratory chain of the mitochondrial OXPHOS complexes as well as 22 tRNAs used in mitochondrial protein synthesis and 2 rRNAs (12 s and 16 s) that are necessary for the translation of mitochondrial subunits [37]. A functionally close and important relationship exists between the nuclear genome and the mitochondrial genome with about 1,500 mitochondrial-related genes that are critical for mitochondrial function residing in the nuclear genome [38–40]. Transcripts for most of the mitochondrial polypeptides are translated in the cytoplasm and imported into the mitochondria. Examples include the mitochondrial transcription factor A (TFAM) [41, 42] that is required for mtDNA replication; and transcription of TFAM is correlated with transcription of catalytic DNA polymerase gamma (POLG) and accessory POLYG2 subunits [43].

In addition to mitochondrial activity being dependent on overall mitochondrial dynamics and motility, mitochondrial activities depend on several specific mitochondrial features and characteristics that are important for cell cycle-specific functions and include the mitochondrial transition pore (mPT), membrane potential, localization and distribution patterns, and adequate amounts of mtDNA. Dysfunctions involve increases in reactive oxygen species (ROS) and calcium overload; reviewed in [3, 8, 11, 12, 14–16].

The mitochondrial permeability transition pore (mPT) is a nonselective and high-conductance channel composed of ANT, the voltage-dependent anion channel (VDAC), and cyclophilin-D [44]. The mPTP is localized to the inner membrane and an increase of mitochondrial inner membrane permeability to ions and solutes with molecular masses up to about 1,500 Da leads to matrix swelling [45]. Changes in the mPTP are important for mitochondrial maturation, as shown in differentiating cardiomyocytes [46].

Mitochondrial membrane potential (Ψm) results from mitochondrial activity, and is highly important for oocyte quality; conversely lowly-polarized mitochondria in oocytes result in abnormal embryos [47]. Several probes are available to determine Ψm and includes the mitochondrial membrane potential indicator 5,5',6,6'-tetrachloro-1,1'3,3'-tetraethylbenzamidazol-carboncyanine (JC-1) [11, 48]. JC-1 is a cationic dye whose mitochondrial uptake is directly related to the level of Ψm. A greater concentration of JC-1 aggregate is correlated with greater mitochondrial uptake resulting in a red fluorescent emission signal; while green fluorescence indicates the presence of JC-1 as monomer. A higher red:green ratio indicates a more polarized, or more negative and hyperpolarized mitochondrial inner membrane. JC-1 has been used to measure mitochondrial maturation and distinguish immature from mature mitochondria.

In somatic cells, mitochondria form a highly complex dynamic network and they undergo fusion and fission to accommodate the functional requirements of specific cell types. In most cell types mitochondria are translocated along microtubules to their functional destinations [49] using the microtubule motor proteins dynein and kinesin for effective intracellular translocations [50]. Mitochondrial locations can change depending on their cell cycle-specific function. In some cases activity is required at specific localized areas in a cell. For example, mitochondria become localized to the nuclear periphery prior to nuclear envelope breakdown. This localization of mitochondria indicates positive developmental potential while mitochondria dispersed in the cytoplasm at the predicted time for nuclear envelope breakdown is indicative of cells or embryos with less developmental potential [49, 51].

**Figure 1:**
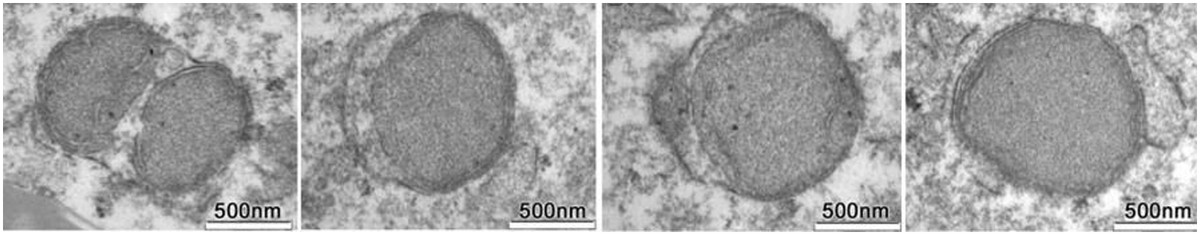
**TEM of mitochondria.** TEM of round mitochondria without cristae in 2-cell stage porcine oocyte at 48 h of in vitro fertilization. From Zhong et al. [4].

As mentioned in the introduction, in contrast to differentiated cells, early embryo cells, like pluripotent stem cells, contain mitochondria that display a round morphology and only few cristae (Figure 1), generally referred to as functionally immature mitochondria and are characteristic of undifferentiated cells. While we do not yet have complete and detailed knowledge about specific mitochondrial functions in embryonic cells we know that mitochondria are important for oocyte maturation, fertilization, and preimplantation development and may contribute ATP for energy-consuming events such as nuclear envelope breakdown, and microtubule assembly and disassembly for meiotic and mitotic spindle assembly.

Oocyte maturation: Mitochondria are critically important for oocyte maturation and they are reliable indicators for oocyte quality achieved during the maturation process. Mammalian oocytes are arrested at the germinal vesicle (GV) stage in the ovary of the newborn which is the diakinesis stage of prophase I. These arrested oocytes remain at the GV stage until puberty, when follicle-stimulating hormone (FSH) induces development of small antral follicles into the pre-ovulatory stage. Oocyte maturation in the ovary continues with the resumption of meiosis from prophase I (germinal vesicle stage; GV) and the extrusion of the first polar body (PBI) followed by initiation of meiosis II and oocyte arrest at the metaphase II stage of second meiosis. The number of mitochondria varies in different species but in most mammalian species mitochondria are closely located around the MII spindle [50, 52]. This localization implies that they are important for spindle formation and maintenance of spindle integrity.

Meiotic spindle formation begins in most mammals at the center of the oocyte (reviewed in [53]) after nuclear envelope breakdown of the germinal vesicle (GVBD). This process of GVBD is triggered through stimulation of the cumulus cells by luteinizing hormone (LH). Perinuclear accumulation of mitochondria is a positive sign of oocyte quality and allows accurate timing of GVBD (reviewed in [53]). The formed spindle migrates to the oocyte cortex and is anchored to the cortex by an actin filament cap (reviewed in [53]).

Oocyte maturation is important for the establishment of oocyte qualities that allow for optimal fertilization and embryo development. During oocyte maturation, the oocyte grows and undergoes remodeling on cellular and molecular levels. This remodeling requires ATP likely supplied by mitochondria; thereby allowing timely and accurate cytoplasmic and nuclear maturation [54]. Mitochondria-supplied ATP is also important for protein phosphorylation and dephosphorylation which are among the regulatory events that play key roles in oocyte maturation, and include centrosome and microtubule dynamics for the formation of the meiotic spindles during meiosis I (MI) and II (MII) [18, 19, 53, 55–57]. Formation of the MI and MII spindle is a critical process to allow accurate chromosome segregation and the two successive asymmetric cell divisions that result in small polar bodies and a large polarized oocyte. The cytoskeleton plays important roles in this cellular and molecular remodeling, and it is essential for accurate redistribution of mitochondria throughout the maturation process [53, 54, 56, 58–61].

The oocyte arrested at the MII stage is the final product of oocyte maturation. On average, the mature mammalian MII oocyte contains approximately 100,000 to 200,000 mtDNA copies [62] and amounts to almost 300,000 in pig oocytes [63]. The number of mtDNA in an oocyte is positively correlated with fertilization- and developmental- competence. Other indicators for positive developmental potential are the distribution of mitochondria surrounding the MII spindle in most mammals and positive staining with Brilliant Cresyl Blue (BCB). The BCB dye can be broken down by glucose-6-phosphate dehydrogenase (G6PD) and can thus be used as an indicator of G6PD activity. BCB has been used reliably to determine oocyte quality [64]. G6PD is synthesized and accumulated during the oocyte growth phase; it is a rate limiting enzyme needed for NADPH production through the PPP. The different levels of BCB indicate variations in oocyte quality as estimated by G6PD. Oocytes with insufficient G6PD to reduce the dye, stain blue and are less likely to be developmentally competent, while oocytes not staining blue contain G6PD and reduce the dye to a colorless solution, indicating that these oocytes may be more developmentally competent. Other indicators for oocyte quality have also been used and have been reviewed in [65, 66].

Functional motor proteins and protein kinases that are needed for meiotic spindle assembly and chromosome alignment require ATP [16], and insufficient ATP production is associated with spindle abnormalities. Both meiotic divisions are error-prone in humans and chromosomal aneuploidies have been reported for the first and for the second meiosis [18, 19, 67–70]. Aberrant functions of motor proteins and kinetochore-related kinases may contribute to chromosomal aneuploidies in females with quality-compromised oocytes [71]. Abnormalities in polo-like kinase (Plk) functions also have been implicated in aneuploidy. Since ATP is required for the function of protein kinases, low kinase activity may result in mitochondrial dysfunction [8, 72, 73].

Fertilization: When the MII oocyte is ovulated it enters the oviduct where fertilization occurs. Fertilization initiates completion of meiosis II and is marked by extrusion of the second polar body (PBII) to achieve the haploid maternal contribution to the oocyte.

Mitochondria are maternally inherited in mammalian embryos, and the MII oocyte contains all mitochondria for subsequent development to the blastocyst stage [74]. The sperm’s mitochondria are destroyed during the fertilization process [75, 76] and the oocyte’s immature mitochondria are distributed equally to the dividing daughter cells during first and subsequent cell divisions. Unequal distribution leads to disproportional patterns of mitochondrial inheritance [77] in 2- to 4-cell stage human embryos resulting in cell lysis of the blastomere that is deficient in mitochondria. Loss of cells from the developing embryo may have negative consequences for embryo implantation.

Preimplantation embryo development: Remodeling of mitochondrial features is important for differentiation when increased energy is needed for embryo development. During preimplantation embryo development several changes take place in mitochondrial architecture as they transform from a simple spherical structure to begin displaying more complex morphologies, including well-developed cristae, a denser matrix, and an elongated or branched appearance. This more complex change in morphology is correlated with a bioenergetic transition from mainly glycolytic to aerobic OXPHOS metabolism. This transition includes an increase in the respiratory chain complex density and ATP production. Several proteins drive the mitochondrial differentiation process which have been determined to some extent [1, 2] but many remain undetermined. As cellular differentiation occurs mitochondrial differences are especially apparent during blastocoel formation when cells are differentiated into trophectoderm (TE) and inner cell mass (ICM) cells [1, 2, 78, 79]. Mitochondrial elongation and increased cristae formation is first observed in TE cells during their differentiation into an epithelial layer [49, 51, 78] while ICM cells retain spherical mitochondria that contain only few or no cristae. The spherical shape in ICM cells is also characteristic for pluripotent stem cells and many cancer cell types.

Many of the studies on maturation, fertilization and embryo development have been performed in animal models such as the mouse, rabbit, bovine, and porcine systems and fewer studies are available for human; although this area of research is progressing more rapidly now because of the need for higher success rates in IVF clinics.

## Section 2: mitochondrial dysfunction in quality-compromised oocytes

As mentioned in section 1, oocyte quality is important for fertilization and development into viable offspring. Quality-compromised oocytes are correlated with infertility, developmental disorders, reduced blastocyst cell number and embryo loss, but the mechanisms underlying these effects are not well understood. Oocyte quality is achieved during the maturation process as addressed in section 1. Maturation defects can have several causes and many have been associated with mitochondrial dysfunction [18, 19] due to insufficient ATP, calcium homeostasis, hormonal effects, and several others.

Several metabolic changes or disorders such as obesity, diabetes, and aging among others, play a role in reduced oocyte quality. Metabolic disorders not only affect oocytes in the adult female but they also affect all oocytes produced in the developing fetus during pregnancy of the mother, as the adverse intrauterine environment directly affects oogenesis in the fetus and the embryo’s primordial germ cells from which the next generation develops [80].

As mentioned in section 1 sufficient oocyte maturation, both nuclear and cytoplasmic, is important to acquire fertilization-competency (reviewed in [18]. Mitochondria are essential for cytoplasmic maturation to contribute ATP (reviewed in [81]) which is needed for critical cytoplasmic and cellular functions [82]. For effective cytoplasmic maturation a minimum amount of mitochondrial DNA (mtDNA) copy number is important; this has been demonstrated for mouse oocytes [83], bovine oocytes [84], porcine oocytes [64] and human oocytes [62]. While the copy numbers in mouse, bovine and porcine oocytes does not vary significantly between individual oocytes of the same species, interestingly, in the human, there is significant variability ranging between 20,000 and 598,000, with a mean of 193,000 [62], mean values of 256,000 [85], 314,000 [86] and mean values of 795,000 [87] copies. This significant range in human oocytes may reflect the range of different IVF patients analyzed, and it may be indicative of the wide range of oocyte quality in humans that may be correlated with fertility and decreased fertility/infertility problems [88]. A threshold level of mtDNA is necessary to support fertilization and embryo development [64, 83]. As all mitochondria are maternally inherited (reviewed in [18, 19]) the threshold number of mitochondria in the metaphase II (MII) oocyte is critical. The regulation of oocyte mDNA copy number has been studied in detail by Mao et al. [63]) and it has been shown that follicular fluid, epidermal growth factor (EGF) and neuroregulin 1 play a significant role during *in vitro* maturation and subsequent embryo development in pigs.

Oocyte aging is strongly associated with mitochondrial dysfunction [6, 8, 13, 17] for which underlying causes have been explored and include: an increase in mtDNA damage with changes in copy number and mutational load; changes in mitochondrial gene expression; a decrease in mitochondrial membrane potential; changes in mitochondrial dynamics; increased density of mitochondrial matrix; frequency of ruptured mitochondrial membranes; a decrease in Δφ_m_, a decrease in ATP synthesis and metabolic reactions in the electron transport chain; and increased production of reactive oxygen species (ROS). The excessive reactive oxygen species (ROS) generation is closely associated with the oxidative energy production or calcium overload, which may trigger opening of the transition pore and subsequent apoptosis.

ROS is generated during the production of ATP and it causes oxidative damage to mitochondrial DNA if not detoxified, which results in mutations and deletions of mtDNA. As repair enzymes for mtDNA are minimal, mitochondria are especially sensitive to oxidative stress-induced damage [89]. The mutation rate in mitochondrial DNA is 10- to 20-fold higher compared with nuclear DNA, and it is likely related to the limited DNA repair capacity [90, 91]. A number of different mitochondrial deletions and mutations have been reported and the most common deletion has been identified in the human mitochondrial genome as a 4,977 bp deletion within two 13 bp repeats, beginning at positions 8,470 and ending at 13,459 [92]. The accumulation of the 4,977 bp deletion within mtDNA is considered a marker for aging [85, 93–95] and is related to lower energy production. Accumulations of mutational loads to the mtDNA adversely affects mitochondrial functions and will negatively impact development of the preimplantation embryo. In addition, oocyte mitochondria with mutations may pass these mutations to the offspring who will inherit the susceptibility to metabolic diseases [96, 97].

Chromosomal aneuploidies are well known effects of oocyte aging but the time-dependent detailed cascades and specific mechanisms leading to loss of spindle integrity and aneuploidy are still being explored. A detailed cause and effect mechanism is not known. However, several aspects are clearly observed during the aging process and include decreases in motor protein functions as addressed above in section 1.

Diabetes and obesity are two of the major causes for mitochondrial dysfunction [3, 11, 12, 98–100] which has significant implications for oocyte quality from women with such metabolic disorders or diseases [3, 10–12, 100–102]. Other dysfunctions related to metabolic disorders are probable but many dysfunctions remain unexplained.

Diabetes and obesity have become major concerns worldwide and have now exceeded the undernourished population with estimated numbers of 1.5 billion and 1 billion, respectively. Diabetes and obesity are both significantly associated with infertility and have contributed to the increase in patients seeking ART procedures in IVF clinics. Many of the mitochondrial dysfunctions that are known for aging oocytes are also seen in oocytes from diabetic and obese females: these include a decrease in membrane potential, reduced blastocyst formation with imbalances in TE and ICM cell numbers resulting in embryo loss, and several others; reviewed in [3, 11, 12, 100, 103].

## Section 3: possibilities to overcome mitochondrial dysfunction in oocytes to improve IVF success rates

One of the most challenging obstacles for achieving successful *in vitro* fertilization (IVF) and embryo development in IVF clinics is the poor quality of mitochondria in oocytes of obese and diabetic women, of women at advanced reproductive ages, and of women with various other metabolic disorders. This limitation has stimulated a number of different approaches to overcome the defects and includes cytoplasmic transfer which refers to the supplementation of an oocyte with donor cytoplasm containing healthy mitochondria (mitochondrial supplementation). While this approach had raised concerns as mentioned in the introduction [26] cytoplasmic transfer has recently been viewed more favorably as an optional therapy to eliminate mitochondrial mutations, thereby allowing the birth of mitochondrial mutation-free healthy children [27–29]. However, there are risks to be taken into account. For example, in ooplasmic transfer, of 13 pregnancies, two fetuses were karyotypically 45, XO (Turner’s syndrome). One of these fetuses aborted spontaneously and the other pregnancy was terminated. This fact is recorded in a FDA document (http://www.fda.gov/OHRMS/DOCKETS/ac/02/briefing/3855B1_01.doc). While different policies exist in different countries [104] and research is not supported by Federal funding agencies clinical applications are possible. The option is available if parents agree to the procedure. With these new considerations [105, 106] new approaches for cytoplasmic transfer into quality-compromised oocytes are possible to overcome mitochondrial dysfunctions or deficiencies. Such procedures are especially important considering that the demand for IVF procedures has increased significantly in recent years. The increase in demand is in part due to the worldwide increases in obesity, diabetes and the trend to have children later in life when oocytes have aged considerably and display mitochondrial insufficiencies [8, 9, 12, 88, 107]. New treatment options for quality-compromised oocytes are needed to overcome insufficient mitochondrial functions.

Because many developmental disorders and diseases are associated with dysfunctional mitochondria (such as neurological diseases [108, 109], diabetes [99, 110] and references therein, cancer [111], the immune system [112] and aging [113], as well as other diseases and disorders that affect a large percent of the population worldwide) research on mitochondria has excelled in recent years. Since mitochondria are versatile and can be manipulated experimentally it is possible to develop new strategies for therapeutic intervention to restore mitochondrial functions once we know the specific defects [36].

In quality-compromised oocytes containing dysfunctional mitochondria transfer of mitochondria-containing cytoplasm from donated fresh oocytes has corrected the mitochondrial lesions [6, 26–29]. The benefits, but also complexities, have been addressed and it was shown that the source of the donor cytoplasm is a major factor in successes or failures. Because of the close functional dependency of mitochondria on the nuclear genome the cell type used for donor transfer is of primary concern and should be closely related to the recipient cell for optimal coordination of mtDNA and nuclear DNA. Ovarian or oocyte-differentiated cells have been shown to yield the highest success rate in transferring donor cytoplasm containing healthy mitochondria into quality-compromised oocytes. Cells of oogonial derivation have been studied in recent years in light of the search for oogonial stem cells or stem-like cells [114–118] for which unresolved controversies exist in the field [119]. However, this research has benefitted the characterization of oogonial-derived cells.

**Figure 2:**
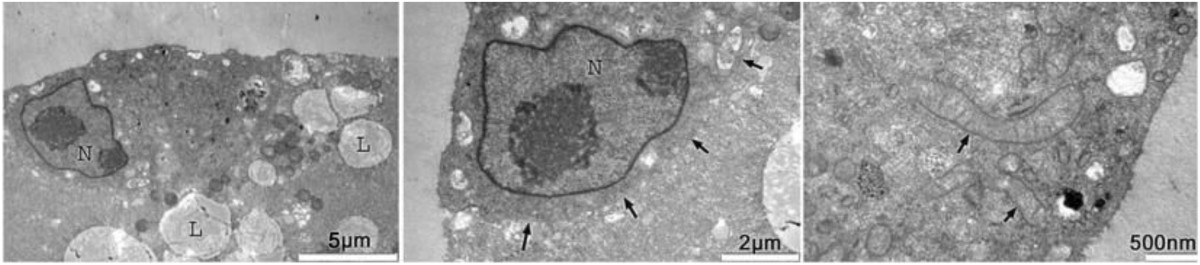
**TEM of mitochondria after SCNT.** TEM of reconstructed (somatic nuclear transfer; SCNT) oocyte at 30 min after fusion. The donor cell is still clearly separated from the enucleated oocyte (delineated by arrows) at this stage of SCNT. These cells contain somatic cell mitochondria with an elongated shape and cristae that co-exist with round oocyte mitochondria for at least up to the blastocyst stages and represent heteroplasmy in the developing pre-implantation embryo. From Zhong et al. [4].

**Figure 3:**
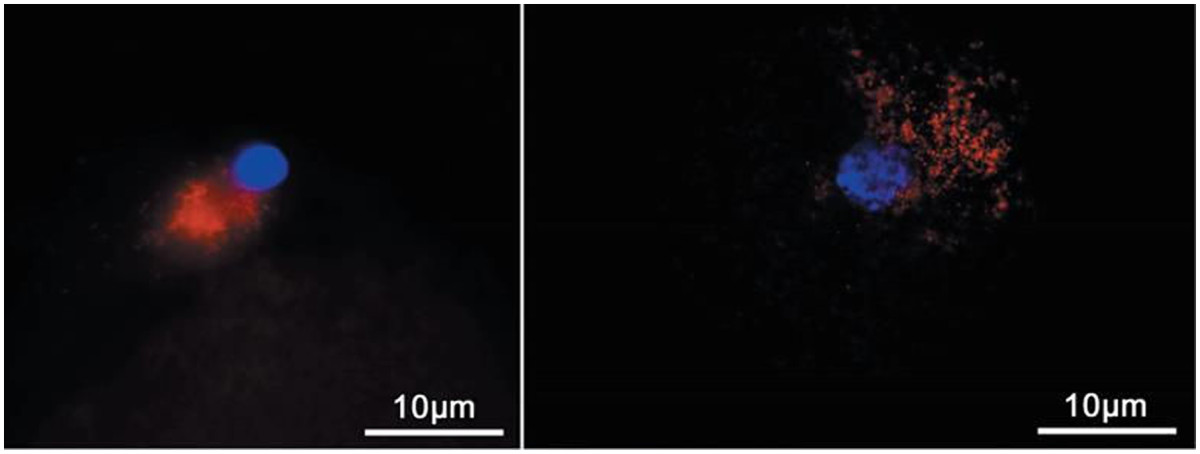
**Fluorescence microscopy of mitochondria after SCNT.** Fluorescence microscopy of donor cell preloaded with CMXRos, 30 min after fusion of donor cell into enucleated oocyte. Donor-derived mitochondria locate near the donor nucleus and disperse into the cytoplasm in developing embryos with heteroplasmy as observed up to the blastocyst stages. From Zhong et al. [4].

**Figure 4:**
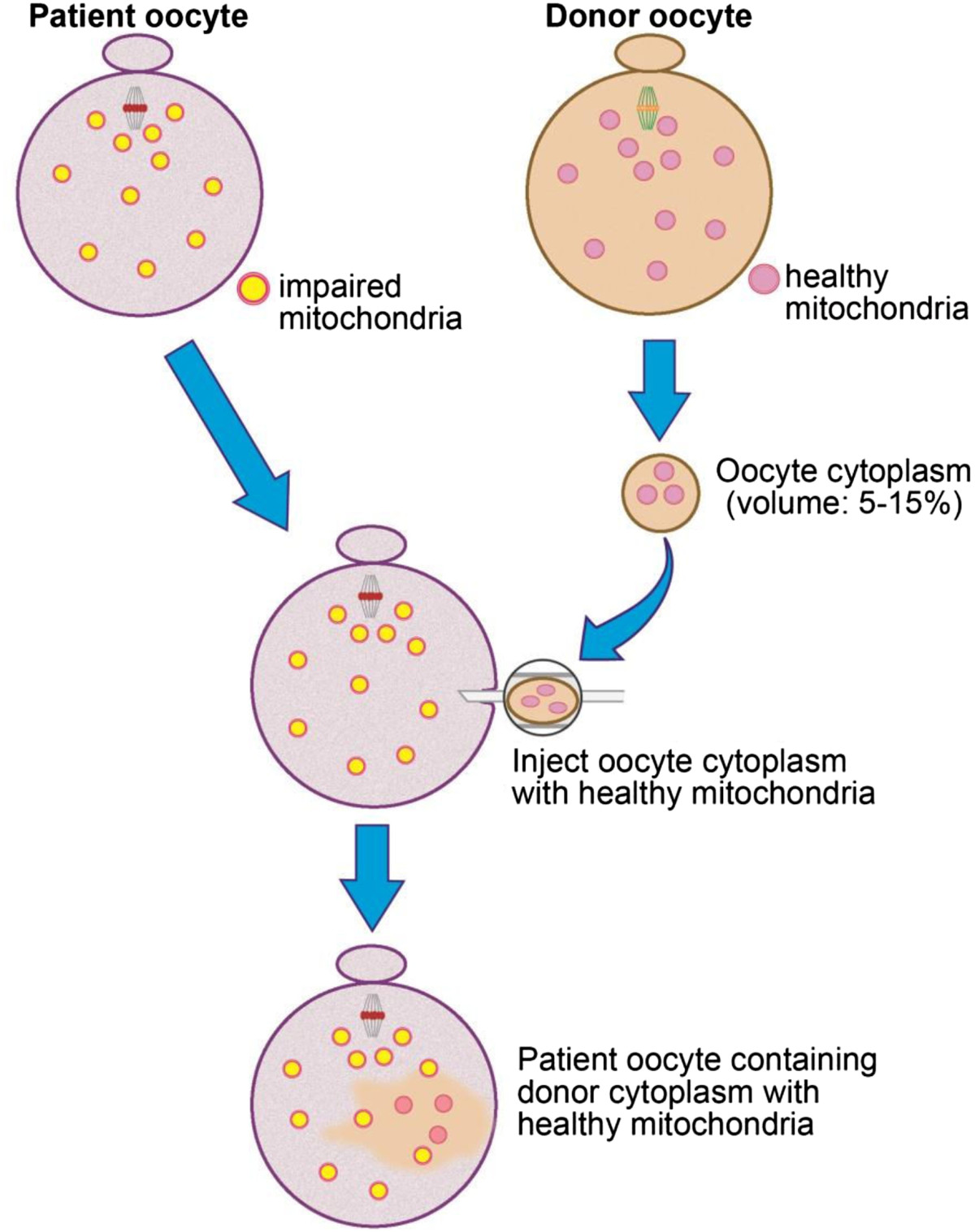
**Schematic diagram of mitochondrial supplementation.** Ooplasmic mitochondrial transfer (schematic diagram). A patient’s oocyte containing inferior mitochondria is injected with donor into the patient’s oocyte to supplement the mitochondrial population required for normal development. From Yabuuchi et al. [122].

Germ line quality mitochondria are optimally available from oocyte precursor cells and may be used preferably to reduce possible compromised oxidative phosphorylation function that may be encountered as a result of mitochondrial heteroplasmy (mixing of different mtDNA genotypes) [64, 120]. Heteroplasmy might be a problem and may cause negative effects but it has also has been shown in somatic cell nuclear transfer (SCNT) experiments that heteroplasmy is tolerable to some extent [121] (Figures 2 and 3). In women with 3% of mtDNA heteroplasmy no negative effects are observed [122] (Figure 4). The possibility of oocyte precursor cells has been reported in both the mouse and human [123–129]. These cells might serve as excellent mitochondria donors as they have differentiated to the oocyte lineage without having resided for prolonged times in a postmitotic state and would fulfill the optimal conditions to provide high-quality germ line autologous homoplasmic mitochondrial DNA without containing mutations and deletions [122].

Considering that numerous mitochondrial mutations, deletions, and nucleotide variations are found in quality-compromised oocytes and blastocysts [130] from women of advanced ages and women with metabolic disorders, such as obesity or diabetes new treatments to augment the healthy mitochondrial population through cytoplasmic transfer from uncompromised cells would not only benefit the mothers to overcome fertility problems but also children of affected mothers who otherwise may inherit mitochondria with deficiencies and suboptimal function. In many cases a low amount of mitochondrial transfer is sufficient to restore optimal mitochondria function and prevent inheritance of suboptimal mitochondria to the next generation.

Other sources for mitochondrial transfer may include stem cell cytoplasm, as stem cell mitochondria display many of the characteristics that are known for embryonic immature mitochondria (reviewed in [1, 7] including the round shapes, glycolysis for energy generation, and amenability to differentiation under different environmental conditions.

Aside from cytoplasmic transfer dietary aspects (such as dietary supplementation with CoQ10 that may increase mitochondrial activity) have also been considered to improve mitochondria functions in quality-compromised oocytes [131]. CoQ10 aids in the transport of electrons in the mitochondrial respiratory chain and is therefore a key enzyme in energy production. Other therapies are being considered and a variety of approaches have been proposed to develop strategies for mitochondrial pharmacology leading to the identification of druggable mitochondrial targets [36].

Different from approaches to supplement mitochondria through cytoplasmic transfer are the approaches that are currently being discussed to eliminate inherited mtDNA mutations. To eliminate the transmission of mtDNA mutations to the offspring two oocytes from different females are needed and several approaches are possible.

In one approach either the GV or the MII spindle and associated chromosomes are removed from the patient’s unfertilized egg containing the abnormal mitochondria and transferred into an enucleated donor egg containing healthy mitochondria. The donor egg now primarily contains the donor’s mitochondrial DNA but the patient’s nuclear DNA although some of the patient’s mitochondrial DNA is transferred along with the nucleus. This reconstituted egg now can be fertilized with the patient’s partner through the intracytoplasmic sperm injection (ICSI) procedure; thereby now containing the future parents’ nuclear DNA but mitochondria from the donor. The cleaving embryo with normal mitochondria and maternal and paternal genomes can then be transplanted into the patient’s uterus. This approach has also been characterized as “three-parent in vitro fertilization” but in fact the nuclear genetic material is only contributed by two parents. It is primarily used to prevent transmission of inherited mitochondria disease. This approach has been discussed extensively during the past year and resulted in approval by the Food and Drug Administration (FDA) [106]. It had previously been evaluated in 2012 by the UK Human Fertilization and Embryology Authority (HFEA) [104]. Details of this procedure are described in several recent papers including [26, 28–31, 122, 132, 133].

In another approach the patient’s egg containing abnormal mitochondria is fertilized with the partner’s sperm and allowed to develop to the patient’s zygote stage that still contains the abnormal mitochondria. The patient’s pronuclei are then removed and transferred to an enucleated donor egg containing healthy mitochondria. The reconstructed zygote is then processed further for transfer into the patient’s uterus.

In a more recent approach the recipient oocyte is enucleated by removing the MII spindle; a donor oocyte is fertilized and the second polar body is removed and then transferred to the recipient oocyte [134], now containing the donor oocyte’s nuclear DNA but minimal amounts of mitochondria, as the polar body only contains a small amount of mitochondria and mainly consists of one chromosome set that is extruded naturally to restore the haploid oocyte condition as detailed in section 1. The second polar body contains the same amount of nuclear DNA as the oocyte. The recipient oocyte with healthy mitochondria now contains the nuclear DNA of the donor and can then be used for IVF following regular IVF procedures. Variations of these current procedures are possible to eliminate transmission of mutant mtDNA to children and will allow the generation of children without inheriting mitochondrial diseases from affected parents.

Other therapeutic approaches are actively being pursued to improve mitochondrial dysfunction and include approaches based on genome editing. For example, specific elimination of mutant mitochondrial genomes in patient-derived cells by mitoTALENs have been reported [135] in which mitochondria-targeted TALEN expression led to permanent reductions in deletion or point-mutant mtDNA in patient-derived cells.

## Perspectives and future directions

The past decade has seen enormous advances in potential therapies for quality-compromised oocytes that will particularly benefit women with metabolic disorders such as obesity, or diseases such as diabetes, or metabolic changes associated with aging. Dietary components such as CoQ10 and especially transfer of mitochondria from cells with mitochondrial integrity have opened new possibilities for therapeutic advances which will increase the success rates for oocytes of women with compromised oocyte quality. Optimal conditions for such transfers include that mitochondrial supplementation should be obtained from the patient’s own cells for optimal communication between mtDNA and nuclear DNA to maintain homoplasmy in offspring; using cells of ovarian or oocyte origin; and using mitochondria of high-quality cells without the risk to transmit deletions or mutations to the offspring. Stem cell mitochondria may be an optimal source for supplementation of quality-compromised oocytes.

## Authors’ original submitted files for images

Below are the links to the authors’ original submitted files for images. Authors’ original file for figure 1 Authors’ original file for figure 2 Authors’ original file for figure 3 Authors’ original file for figure 4
