# Supplementary figures and images for: The impact of mitochondrial function/dysfunction on IVF and new treatment possibilities for infertility
Source: Reprod Biol Endocrinol. 2014 Nov 24;12:111. doi: 10.1186/1477-7827-12-111 (PMC4297407; doi:10.1186/1477-7827-12-111)

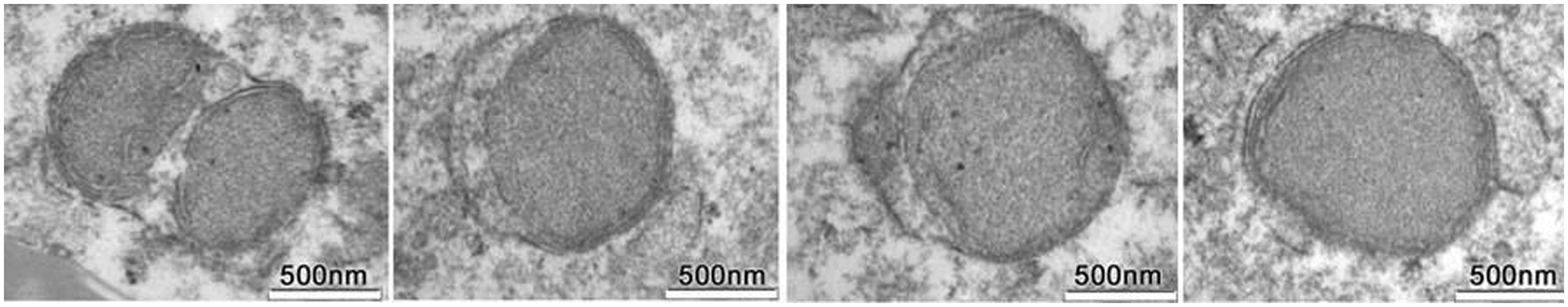

Supplement: Supplementary file 1 — Authors’ original file for figure 1 [file 12958_2014_1300_MOESM1_ESM.tif]

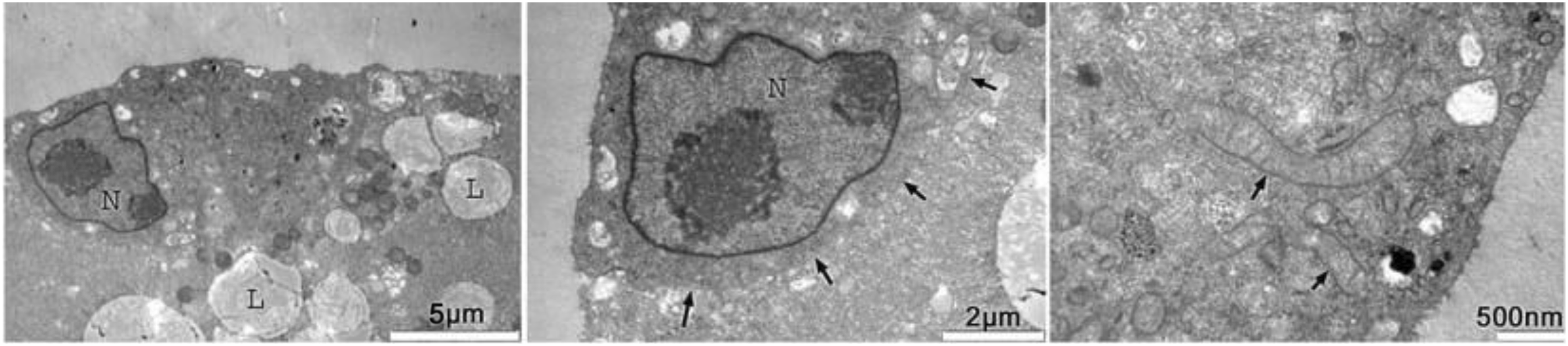

Supplement: Supplementary file 2 — Authors’ original file for figure 2 [file 12958_2014_1300_MOESM2_ESM.tif]

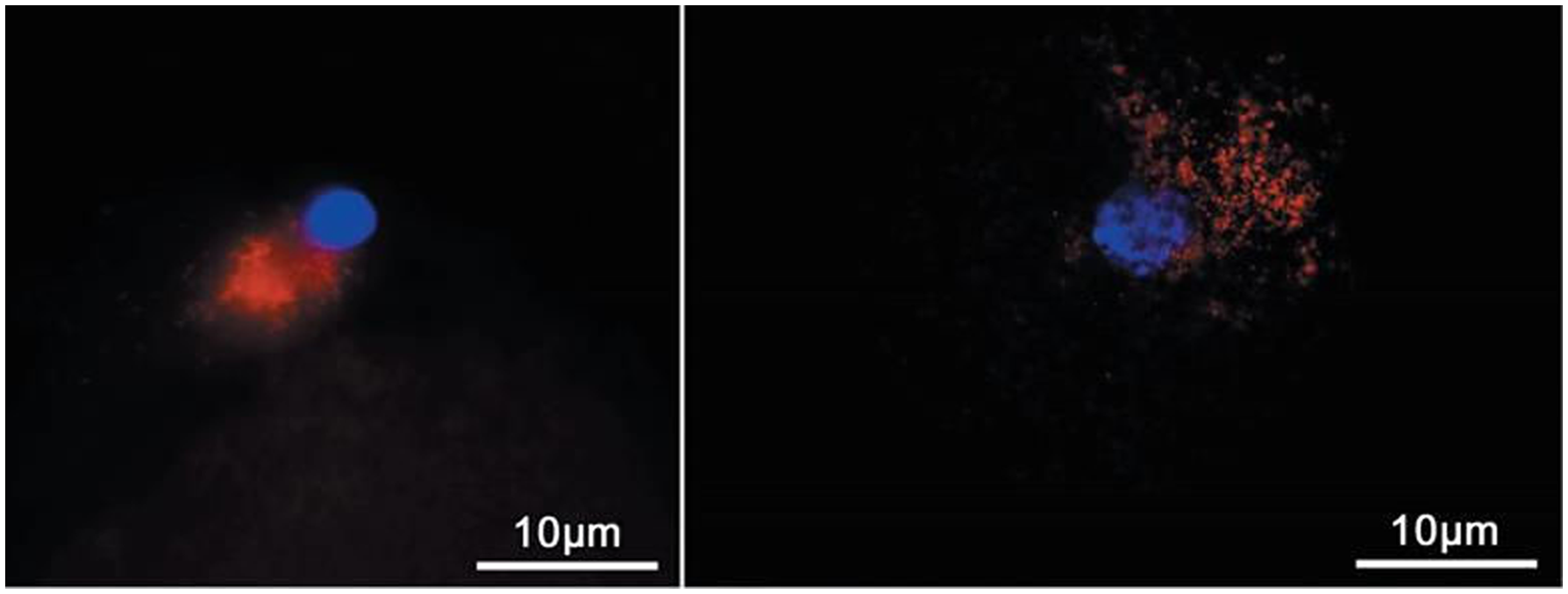

Supplement: Supplementary file 3 — Authors’ original file for figure 3 [file 12958_2014_1300_MOESM3_ESM.tif]

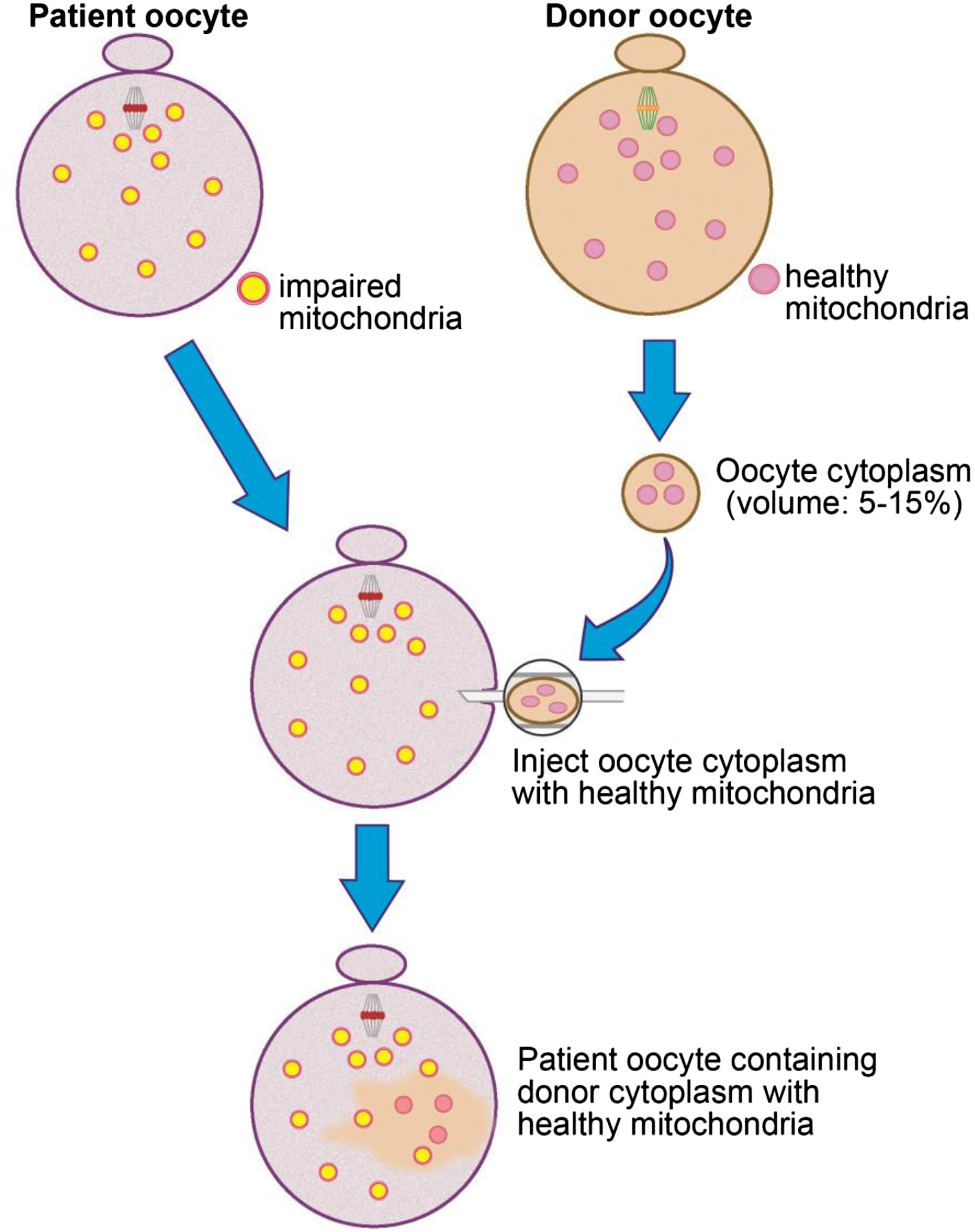

Supplement: Supplementary file 4 — Authors’ original file for figure 4 [file 12958_2014_1300_MOESM4_ESM.tif]
